# ART adherence changes among patients in community substance use treatment: a preliminary analysis from MACH14

**DOI:** 10.1186/1742-6405-9-30

**Published:** 2012-10-11

**Authors:** Marc I Rosen, Anne C Black, Julia H Arnsten, Jane M Simoni, Glann J Wagner, Kathleen Goggin, Robert H Remien, Carol E Golin, Yan Wang, David Bangsberg, Honghu H Liu

**Affiliations:** 1 Department of Psychiatry, Yale University School of Medicine, New Haven, CT; VA Connecticut Healthcare System, West Haven, CT, 06516, USA; 2 Departments of Medicine and Psychiatry & Behavioral Sciences, Albert Einstein College of Medicine and Montefiore Medical Center, Einstein/Montefiore Center for AIDS Research, Bronx, NY, 10467, USA; 3 Department of Psychology, University of Washington, Seattle, WA, 98195, USA; 4 Rand Corporation Health Unit, Santa Monica, CA, 90407, USA; 5 Department of Psychology, University of Missouri-Kansas City, Kansas City, MO, 64110, USA; 6 HIV Center for Clinical and Behavioral Studies, NY State Psychiatric Institute and Columbia University, New York, NY, 10032, USA; 7 UNC Departments of Medicine and Health Behavior & Health Education; UNC Center for AIDS Research, Cecil G. Sheps Center for Health Services Research, Chapel Hill, NC, 27599, USA; 8 Department of Biostatistics, University of California Los Angeles (UCLA), Los Angeles, CA, 90095, USA; 9 Mbarara University of Science and Technology; Harvard Medical School; Ragon Institute of MGH, Harvard, and MIT; Division of Global Health Equity at Brigham’s and Women’s Hospital; Massachusetts General Hospital Center for Global Health, Boston, MA, 02114, USA; 10 Schools of Dentistry, Medicine and Public Health, University of California Los Angeles (UCLA), Los Angeles, CA, 90095, USA

**Keywords:** Medication adherence, AIDS, Substance abuse, Treatment

## Abstract

**Background:**

Opiate substitution treatment has been associated with better adherence to lifesaving antiretroviral medications, but the impact of other substance abuse treatment on adherence is unknown.

**Findings:**

In this study, 215 patients who had been in adherence-focused research studies provided electronically-measured adherence data and a measure of whether the patient had recently been in substance abuse treatment. Recent engagement in substance abuse treatment was independently associated with significantly higher adherence, after covarying for recent substance use and other factors potentially affecting adherence.

**Conclusions:**

The findings suggest that substance abuse treatment is associated with better adherence. Potential mechanisms by which substance abuse treatment improves adherence, such as more stability or more future-orientation, require further study.

## Background

There are considerable data suggesting that current substance abuse is associated with both worse adherence to antiretroviral medications and worse attendance at HIV treatment visits [1]. HIV-positive patients who use drugs or alcohol may be referred to standard substance abuse treatment with the hope that medication adherence will improve with abstinence.

However, among substance abuse treatments, only opiate substitution treatment has consistently been associated with better adherence, as summarized in a 2008 meta-analysis [2]. Both buprenorphine [3,4] and methadone maintenance [5,6] have been consistently associated with better adherence. Patients who received both opiate substitution treatment and directly observed therapy have shown even better adherence over time than with those receiving opiate substitution alone [7,8].

It is not known whether enrollment in substance abuse treatments other than opioid substitution is associated with better adherence. In this study, we used data pooled from two clinical trials of adherence-focused interventions to test the hypotheses that HIV-infected people who had recently been in substance abuse treatment would have better antiretroviral adherence, and better adherence over time, than those who had not had recent substance abuse treatment.

## Methods

### Overview and sampling

The data were drawn from two of the sixteen studies in the MACH14 cohort [9]. The MACH 14 cohort is a dataset pooled from 16 studies conducted at 14 sites across 12 states. Each study in MACH14 used electronic data monitoring (EDM) pillcaps to objectively measure participants’ adherence to antiretroviral medication. The focus of this study was on non-methadone substance abuse treatment so studies conducted in methadone maintenance programs were not considered in this analysis. From the 1579 participants in the MACH14 dataset, we identified 215 from two studies based outside methadone clinics [10,11] because only these two studies’ participants had both EDM and substance abuse treatment status data. Written informed consent was obtained for participation in the parent studies, and the Yale Institutional Review Board approved the secondary analyses.

Patients were asked about engagement in substance abuse treatment and use of specific substances for varying preceding timeframes: one of the two studies (n=47) asked about participation in substance abuse treatment during the past 90 days and use of specific substances over the past 30 days, while the other study (n = 168) asked about treatment over the past 30 days and substance use over past 14 days. To aggregate substance use data across studies, variables representing use of specific substances were defined as the proportion of days within the asked-about timeframe the person had used each of several substances.

This analysis used data collected at the first time-point at which participants had EDM data for the preceding four weeks, had also been asked about being recently enrolled in substance abuse treatment, and were not enrolled in a methadone-clinic-based study. To estimate the effect of substance abuse treatment on adherence, adherence was calculated for the four weeks up to and including the date recent substance abuse treatment enrollment was assessed, as well as for the four weeks after the substance abuse treatment determination. Adherence in each week was calculated by dividing the weekly number of doses taken by the weekly number of prescribed doses for each medication, with adherence to each medication capped at 100%. Adherence for a patient on multiple anti-retrovirals was calculated by averaging across prescribed medications.

### Data analysis

The effects of substance abuse treatment on adherence were determined in multivariate analyses that included a grouping variable denoting whether the patient was enrolled in substance abuse treatment and a variable reflecting substance abuse treatment over time. The analyses were conducted controlling for sociodemographic characteristics that might differ between patients in, and not in, substance abuse treatment. To control for the anticipated finding that patients in substance abuse treatment would have more active drug use than a reference group including people who had *never* had significant substance use, analyses included a measure representing the largest proportion of days during which participants had used either cocaine, opiates, or stimulants. Cannabis use was not included in this measure of illicit drug use because in a separate analysis of the MACH14 dataset [12] and in an earlier study [13] recent cannabis use was not associated with worse adherence.

Analyses were run with SAS 9.2. The model included random effects for intercept and slope as this model had better fit to the data than models with fixed effects only.

## Results

### Baseline characteristics

Overall, the sample was 65.7% male, and was composed of 49.3% African American, 2.8% Hispanic, and 19.5% Caucasian participants. Only 40.1% were employed. Altogether, 39.5% were men who have sex with men (MSM). Mean CD4+ count was 232.2 (+/− 206.9) and 8.5% of the 199 patients with viral load results available had undetectable HIV viral loads (<400); these measures of HIV disease did not differ between those in and those not in substance abuse treatment. Mean adherence during the first week was 82% (+/− 30%) overall, with 57.2% (123/215) having 100% adherence, and 12.6% (27/215) having adherence over 90% but less than 100%.

Of the 215 participants, 13 had recently been in substance abuse treatment--- one had been in residential treatment and the rest did not specify the type of treatment attended. Table 1 compares those in substance abuse treatment to those not in it; those in treatment were less likely to be African American (Fisher’s Exact Test =.04), and had used alcohol on a lower percentage of days (t=5.9, p<.0001). Only one of the thirteen patients in substance abuse treatment had used alcohol recently and none had used other substances, whereas there were substantial rates of recent use of several substances among those out of treatment.

**Table 1:** Baseline group characteristics

	**In Substance Abuse Treatment**		**NOT In Substance Abuse Treatment**		**Test Statistic**	**df**	**p**
	**N**	**mean +/− sd or %**	**N**	**mean +/− sd or %**			
**Demographic Variables**	13		202				
Age	13	38.8+/−8.0	187	40.3+/−8.2	t=−0.63	198	NS
Gender (Male)	9	69.2%	131	65.5%	= 0.08	1	NS
Ethnicity					Fisher Exact		0.04
Caucasian	5	38.5%	37	18.3%			
African American	2	15.4%	104	51.5%			
Hispanic	0	0%	6	3.0%			
Employed	2	18.2%	65	41.7%	Fisher Exact		NS
MSM	5	55.6%	80	63.5%	Fisher Exact		NS
**HIV Markers**							
Mean CD4+	13	295.4+/−202.6	186	227.8+/−207.0	t=1.14	197	NS
Non-Detectable (%)	12	92.3%	170	91.4%	Fisher Exact		NS
**Substance Use Variables**							
Proportion Days Used Alcohol	13	0.0055+/−0.020	188	0.088+/−0.1775	t=−5.86	173.0	<.0001
% Recent Alcohol Use	1	7.7%	78	38.6%	Fisher Exact		0.034
% Recent Cannabis	0		21	13.4%			
% Recent Cocaine	0		7	3.5%			
% Recent Heroin	0		2	0.99%			
% Recent Stimulants	0		9	4.5%			
% Used Any Illicit Drug	0		17	8.4%			

### Multivariate analysis

The multivariate analysis estimated the effects of sociodemographic measures (age, gender, race/ethnicity, MSM-status, employment), time in weeks, and maximum proportion days reported using an illicit drug (Table 2). As expected, adherence was slightly and significantly worse over time (estimate =−.02, p=.002). Being in substance abuse treatment was associated with better overall adherence (estimate =0.09, p= .05). The week by treatment interaction was not statistically significant and was removed from the model; substance abuse treatment status did not significantly change adherence over time.

**Table 2:** **Impact of Predictors on Antiretroviral Adherence**[1]

**Effect**	**Estimate**	**Pr >|t|**
**Intercept**	0.82	<.0001
**Week**	−0.02	0.0022
**Recent Substance Abuse Treatment (yes/no)**	**0.09**	**0.0471**
**Male**	0.08	0.0216
**African American(Caucasian is Reference)**	0.05	NS
**Hispanic**	0.07	NS
**Other Race**	0.05	NS
**Illicit Drug Use (largest proportion of days reported use of illicit drug other than cannabis)**	−0.13	NS
**Man who has Sex with Men**	0.02	NS
**Employed**	−0.03	NS

1-Adherence was proportion of prescribed doses taken during an eight week period comprising each of 4 weeks before and 4 weeks after substance abuse treatment status was inquired about.

## Discussion

Substance use treatment may improve adherence by several mechanisms. Although the analyses controlled for illicit drug use, it is possible that our self-report measures of substance use understated the impact of substance abuse treatment on substance abuse and that it is in fact abstinence that facilitates adherence. In one of the few randomized controlled studies of HIV-positive drug users in which abstinence was the target outcome, there was a trend towards a significant correlation between consecutive weeks of toxicology-tested abstinence during the intervention and reductions in viral load [14]. There is also evidence from a naturalistic longitudinal cohort study that attendance at HIV treatment, a sine qua non for adherence, appears to improve with newly-achieved abstinence [1].

Substance abuse treatment might improve adherence by mechanisms other than facilitating abstinence from using drugs. Substance abuse treatment typically involves case management [15] to address the unstable housing characteristic of drug users [16]. Stable housing arrangements during substance abuse treatment would be expected to foster adherence, in that stable routines have been associated with better adherence [17]. Substance abuse treatment also focuses patients on future goals, an orientation that has been described as fostering adherence [18], and substance abuse treatment can involve re-arranging social networks in ways that also might foster better adherence [19].

It is possible that enrollment in substance abuse treatment reflects a lurking un-measured variable associated with both being in substance abuse treatment and better adherence. The finding of better adherence among people in substance abuse treatment was not buttressed by finding better adherence *over time* among patients in treatment. However, it might have been difficult to detect the time course of benefit from substance abuse treatment because the data did not specify when patients were entering, continuing, or finishing substance abuse treatment.

There are several other caveats in interpreting these findings. Substance abuse was measured by self-report, and it is possible that substance abuse was disproportionately under-reported by people out of substance abuse treatment, thus exaggerating the impact of substance abuse treatment on adherence. The type of substance abuse treatment was not specified and the findings may not apply to all types of substance abuse treatment. Finally, the sample size was modest, and the number of participants in substance abuse treatment was small. It is noteworthy that although adherence decreased on average over time, the course of adherence varied significantly by person. Further analyses should test variables that may account for individual differences in adherence over time.

These findings lend some support to the clinical practice of addressing substance use in an effort to improve adherence. The crucial next step is to develop and prospectively test substance abuse-focused interventions for patients with both substance abuse and adherence problems.

## Competing interests

The authors declare that they have no competing interests.

## Authors' contributions

MR designed the analyses and drafted the manuscript. All the authors read and approved the final manuscript. All the authors except ACB and YW contributed patient data to the MACH 14 dataset. ACB helped design the analyses, and the aggregated dataset from which they were drawn. YW and HHL conducted the statistical analyses.
